# Climate change-driven elevational changes among boreal nocturnal moths

**DOI:** 10.1007/s00442-020-04632-w

**Published:** 2020-04-08

**Authors:** Netta M. Keret, Marko J. Mutanen, Markku I. Orell, Juhani H. Itämies, Panu M. Välimäki

**Affiliations:** 1 Ecology and Genetics Research Unit, University of Oulu, PO Box 3000, FI-90014 Oulu, Finland; 2 Present Address: Kaitoväylä 25 A 6, FI-90570 Oulu, Finland

**Keywords:** Season length, Phenology, Altitude, Lepidoptera, Temporal trends, Flight period

## Abstract

Climate change has shifted geographical ranges of species northwards or to higher altitudes on elevational gradients. These changes have been associated with increases in ambient temperatures. For ectotherms in seasonal environments, however, life history theory relies largely on the length of summer, which varies somewhat independently of ambient temperature per se. Extension of summer reduces seasonal time constraints and enables species to establish in new areas as a result of over-wintering stage reaching in due time. The reduction of time constraints is also predicted to prolong organisms’ breeding season when reproductive potential is under selection. We studied temporal change in the summer length and its effect on species’ performance by combining long-term data on the occurrence and abundance of nocturnal moths with weather conditions in a boreal location at Värriötunturi fell in NE Finland. We found that summers have lengthened on average 5 days per decade from the late 1970s, profoundly due to increasing delays in the onset of winters. Moth abundance increased with increasing season length a year before. Most of the species occurrences expanded upwards in elevation. Moth communities in low elevation pine heath forest and middle elevation mountain birch forest have become inseparable. Yet, the flight periods have remained unchanged, probably due to unpredictable variation in proximate conditions (weather) that hinders life histories from selection. We conclude that climate change-driven changes in the season length have potential to affect species’ ranges and affect the structure of insect assemblages, which may contribute to alteration of ecosystem-level processes.

**Electronic supplementary material:**

The online version of this article (10.1007/s00442-020-04632-w) contains supplementary material, which is available to authorized users.

## Introduction

As a response to global climate warming, a taxonomically wide range of organisms have reacted to derived environmental changes by shifting or expanding their distributions to higher latitudes and/or altitudes (Parmesan 2006; Parmesan et al. 1999; Walther et al. 2002; Root et al. 2003; Hickling et al. 2005; Fox et al. 2014; Nieto-Sánchez et al. 2015; Fält-Nordmann et al. 2018). The phenomenon is particularly strong among ectotherms and in mountainous regions because insect physiology and performance depend on temperature (Angilletta 2009) and elevational gradients allow species to track changing conditions over reasonably short distances (Pounds et al. 1999; Hill et al. 2002; Peñuelas and Boada 2003; Wilson et al. 2005; Franco et al. 2006; Merrill et al. 2008; Chen et al. 2009). Species’ distribution is not only determined by abiotic but also by biotic environments. Herbivorous insects, for example, cannot expand beyond geographic ranges of certain host plants that permit high juvenile performance although the prevailing climate would sustain population persistence (Merrill et al. 2008). This explains why generalist species appear to be the main beneficiaries of warming climates (Warren et al. 2001; Menéndez et al. 2006, 2007; González-Megías et al. 2006, 2008; Pöyry et al. 2009; Clavel et al. 2010). Indeed, recent changes in species’ ranges have modified structures of certain insect assemblages as a whole (González-Megías et al. 2006, 2008; Chen et al. 2009; Zografou et al. 2014; Nieto-Sánchez et al. 2015).

Temperate insects at high latitudes and altitudes face a range of external conditions that constrain individual fitness, such as low ambient temperatures and short favorable seasons for growth and reproduction (Angilletta 2009). Given that ambient temperature affects insect body size, the warming climate may affect fitness in a predictable manner; increasing temperature increases body size, and thus fecundity (see Honěk 1993), until a certain point beyond which an opposite pattern emerges (Davidowitz and Nijhout 2004). A perspective that an increase in ambient temperature as such would underlie climate change responses across species is, however, overly simple and presumably applicable to species that show pronounced thermal determination of life histories, i.e., species with short generation times and multiple generations within a season (Chown and Gaston 1999; Blanckenhorn and Demont 2004). Rather, a theory of insect life history evolution that explains variation in fitness in seasonal environments is grounded upon variation in the length of favorable season than ambient temperature per se (Roff 1980; Iwasa et al. 1994; Kivelä et al. 2013). Season length represents an ultimate constraint; an individual has to reach a specific developmental stage that is capable of diapause to survive the forthcoming winter (Tauber et al. 1986; Angilletta 2009). A mismatch between generation time and season length constrains fitness and may thus affect geographic distribution of insects (Van Dyck et al. 2015). Still, the central role of season length in theoretical considerations is either obscured or only implicitly acknowledged in empirical attempts to understand implications of the ongoing climate warming for insect population dynamics and range modifications.

Latitudinal and altitudinal changes in the environment are expected to result in genetically determined clinal variation in life histories. When the season length becomes sufficiently long, an increase in the number of generations within a year is predicted (Roff 1980; Iwasa et al. 1994; Kivelä et al. 2013). Within a certain degree of voltinism, increasing season length relaxes selection for early maturation and high initial reproductive effort, which enables a long adult life span and high life time fecundity (Kivelä et al. 2013). Assuming a constant adult mortality, a long adult lifespan would be attributable to a prolonged flight period of a particular species. These predictions are applicable to temporal trends as long as season length changes over time. Empirical evidence of prolonged adult periods is reported, although possibly confounded by simultaneous increase in the expression of multivoltinism (Roy and Sparks 2000; Pöyry et al. 2011). Exploration of these variables within a strictly univoltine region would unambiguously avoid collinearity between the pattern of voltinism and duration of adult period.

It is implicitly assumed that increasing temperature as such underlies changes in species’ distribution. We argue that the causality between the habitable climatic envelope and temperature is not straightforward but dependent on a correlated change in the season length, a key factor of theoretical considerations of insect life history evolution in seasonal environments (Roff 1980; Iwasa et al. 1994; Kivelä et al. 2013). The effect of season length on insect population dynamics is not explicitly accounted for thus far, which undermines the theoretical background of many earlier contributions on the implications of recent climate warming. By combining long-term data on nocturnal moth (Lepidoptera) catches with climatic variables from a boreal location, we explored if the season length has increased over time and if the season length has a capacity to constrain insect fitness. The latter would be indicated by a positive correlation between moth abundance and season length a year before. Second, we tested temporal trends in mean elevation values (i.e., elevational center-of-gravity) among strictly univoltine moth species with adequate data and explored ecological dimensions of variation in species-specific responses. The approach is warranted because the historical prewarming distributions of different taxonomic groups may vary and species with different habitat associations, dispersal capacities or thermal physiologies (Thomas et al. 2001; Warren et al. 2001; Hill et al. 2002; Menéndez et al. 2006; González-Megías et al. 2006, 2008; Pöyry et al. 2009; Clavel et al. 2010) as well as their host plants (Kullman 2002; Maliniemi et al. 2018; Franke et al. 2019) might show different responses to changing climate. Third, we analyzed if flight periods of the most abundant species have prolonged in time, which would be consistent with the theoretical prediction based on relaxing seasonal time constraints (Kivelä et al. 2013) and ambiguous empirical evidence (Roy and Sparks 2000; Pöyry et al. 2011). Finally, we explored temporal changes at the community level to evaluate the potential of species level responses in modifying direct and indirect species interactions.

## Materials and methods

### Study site

Värriö Strict Nature Reserve is located in NE Finland (67° 44′ N, 29° 37′ E), ca. 100 km north of the Arctic Circle. The area has remained pristine without anthropogenic disturbance such as settlement, roads or forestry, except a long tradition of reindeer husbandry. The area is in the boreal zone; the snow-free period lasts approximately from the end of May to mid-October. The average temperatures in January (the coldest month) and July (the warmest month) are − 11.4 °C and + 13 °C, respectively. Annual precipitation averages 595 mm. A period of continuous daylight when the sun does not set below the horizon lasts from 30 May to 14 July.

### Moth sampling

For long-term monitoring of nocturnal moths, a transect of 11 light traps (Jalas 1975) ranging from 340 to 470 m a.s.l. on the north-facing slope of Värriötunturi fell was established in 1978 (Pulliainen and Itämies 1988). Three traps were located in a ravine of Norwegian spruce (*Picea abies*)-dominated forest (340–344 m a.s.l.), three traps in an old-growth Scots pine (*Pinus sylvestris*) forest (360–366 m a.s.l.), three traps in a mountain birch (*Betula pubescens* ssp. *czerepanovii*) forest (385–418 m a.s.l.), and two traps on the treeless summit of the fell (462–470 m a.s.l.). Each trap was provided with a 500 W mixed light bulb that was switched on between 20:00 and 08:00 h each night from mid-May to mid-October 1978–2012. Catches were collected each morning. Captured moths were identified by species and counted.

The total moth catch (1978–2012) consisted of 417, 631 individuals representing 463 species. Data were reduced to the most abundant species for analysis to avoid multiple consecutive years of zero catches. We first excluded species that were not resident at the time the survey was started (years 1978–1979) and each regular seasonal migrant, as we were particularly interested in temporal changes in spatial distribution of the resident fauna. From the resident species, we selected the ones that were observed at least in 30 different years with a mean annual catch of more than seven individuals. The selected 57 species were divided among 18 families (Online Resource 1) and comprised 376, 329 individuals (ca. 90% of the total catch). We acknowledge that data reflect measures of abundance and activity of the species collected rather than their population densities in a strict sense. This does not pose severe interpretational ambiguities as our analyses concern temporal changes in moth abundance and phenology among years, and there is no reason to assume that trapping efficiency would significantly change within a species over time (see “Discussion”).

### Data analysis

Data analyses were conducted in R 3.2.3 environment (R Development 2015).

### Temporal trends in timing of the favorable season

The Finnish Meteorological Institute has operated synoptic climate measurements at Värriö Subarctic Research Station since 1971. Manual measurements conducted twice a day by the station staff were replaced by an automatic weather station in the 1990s. The station is located at 360 m a.s.l.; the closest light trap is only a few tens of meters away from the weather station. To characterize temporal trends in climate, we generated three climate variables: “season start”, “season length” and “season end” for years 1977–2013. The “season length” specific to a particular year was estimated as the number of days when average daily temperature had been either above or equal to 10 °C. The threshold effectively characterizes the period available for insect growth and development as temperatures this high coincide with larval or pupal periods of most of the studied species and insect growth practically ceases below the threshold due to temperature dependence of insect development rate (Angilletta 2009). A generalized additive model (GAM) [function *gam* (Wood 2006)] was fitted to the data on average daily temperatures in a particular year to determine robust year-specific estimates for the first and the last date the temperature threshold exceeded. Generalized additive models with the threshold values of 8 °C and 3 °C were then fitted to the year-specific weather data to determine dates for the “season start” and “season end” in Julian days, respectively. These thresholds represented mean daily temperatures across the 35-year study period when the first moth was captured in spring or the last one in autumn. In addition to the biological relevance, underlying rationale of using different threshold values was to avoid strong correlation between any two climate variables.

To explore temporal trends in the climate variables, we regressed the “season start”, “season length” and “season end” against the year [function *lm* (R Development Team 2015)]. Temporal autocorrelations were all weak or moderate (|*ρ*| < 0.35) [function *durbinWatsonTest* (Fox and Weisberg 2011)], thus the model assumption of independent residuals was not violated. The “season length” and the “season end” expressed significant temporal trends (see "Results") and were thus used to explain variance in annual moth catch. The estimates of “season length” and “season end” were not correlated (*r *= 0.098, *t* = 0.583, *df *= 35, *p* = 0.564).

### Bayesian approach

To explore effects of the climate variables on moth population dynamics, species-specific elevation changes and shifts in flight periods across the years, we applied generalized linear mixed-effect models (GLMM) fitted with a Bayesian Markov chain Monte Carlo sampler [function *MCMCglmm* (Hadfield 2010)]. We used inverse-Gamma as required prior distributions of the analysis-specific variance components, the priors being set uninformative by a low degree of belief parameters (*V* = 1, *υ *= 0.002). In the case of random regression models, we used uninformative inverse-Wishart distributions as priors (*υ *= 1.002). The priors were parameterized to approximate to inverse-Gamma (0.001, 0.001) on variances and to some extent Beta (0.001, 0.001) on correlations. Prior sensitivity was analyzed by fitting the same models, but parameterizing the priors so that the observed variance would divide evenly among the variance components with a slight increase in the degree of belief (*υ *= *k* + 1, where *k* is the number of random factors) (Gelman 2006). The results were insensitive to prior assumptions and thus only those based on our primary parameterization are reported. In each analysis, a total of 2,500,000 MCMC iterations were run with a burn-in period of 50,000 iterations. The remaining iterations were sampled with a thinning interval of 100. Upper and lower limits of 95% highest posterior density (HPD) credibility intervals for parameter estimates were derived with the function HPDinterval (Plummer et al. 2006).

#### Effects of the climate variables on moth abundance

To explore if the climate variables that show temporal trends across the study period potentially affect population dynamics of moths, we fitted a GLMM to data on annual moth catch (i.e., abundance). In the model, log-transformed annual catch of a particular species in a particular trap [log^10^(*n *+ 1)] was set as the response variable, whereas fixed effects were estimated for the season length and season end in the preceding year and for elevation and the two-way interactions between the climate variables and elevation. The interaction terms were generated because we were not only interested in the main effects of the climate variables but also if the response among moths varies across the elevational gradient. To increase statistical power of the analysis, elevation was considered as a categorical factor with four levels [spruce forest (340–344 m a.s.l.), Scots pine forest (360–366 m a.s.l.), mountain birch forest (385–418 m a.s.l.), and treeless tundra (462–470 m a.s.l.)], whereas individual traps were considered as repeated measurements within a particular elevation and included as a random factor in the model (*υ*_trap_ = 0.002). The random effect structure included also random intercepts for year and species that was correlated with elevation (*υ*_year_ = 0.002, *υ*_elevation:species_ = 1.002, *υ*_residual_ = 0.002). For species-specific random slope estimation, elevation was considered a continuous variable ranging from 0 to 130 m (340–470 m a.s.l.). Thus, random effect structure took into account year-specific random variation in moth abundance not attributable to the climate variables a year before and species-specific abundances at various elevations.

#### Species’ elevation changes across years

To explore species’ elevation changes (i.e., changes in elevational “center-of-gravity”) during the study period, we fitted a GLMM to data where each individual was considered as an independent observation with a unique elevation value derived from altitude of a trap that had captured it. Variation in individual elevation values was explained by a model with only a fixed intercept that translates altitude of a trap into elevation value of an individual. Species-specific responses were estimated by a random effect structure that included random intercepts for the species that were correlated with year (*υ*_year:species_ = 1.002, *υ*_residual_ = 0.002). This approach approximates to estimation of the elevational "center-of-gravity" for the 57 species for each year that is then regressed against the year (i.e., slope value). The approach is particularly well suited in this case as the studied elevational range is rather limited and thus any trap may occasionally capture some individuals of any species.

To explain variation in species-specific responses (see “Results”), we fitted a GLMM with species-specific slope values as the response variable and a combination of species characteristics (diapause stage, life cycle, host specificity) and ecological factors (host plant characteristics) as well as historical range as the fixed factors (Online Resource 1). Diapause stage was considered a factor with four levels (adult, egg, adolescent larva, fully grown larva or pupa). Species whose fully grown larvae hibernate but do not continue feeding in spring after over-wintering were grouped together with species that over-winter in the pupal stage. This is justified as over-wintering fully grown larvae that complete their growth in autumn, face the same seasonal time constraints as larvae heading for autumnal pupation. The species were classified by their life cycle into two groups that follow either annual or biennial life cycle. The rationale of this division is that species that need to complete their development within a year or season (annual) are more likely affected by seasonal time constraints than the ones that follow a 2-year life cycle (biennial). Host plant growth form was considered a factor with six levels and defined as follows: dwarf shrubs (including deciduous and evergreen species), herbaceous plants, mixed diet (woody dwarf shrubs and herbaceous plants), trees or bushes (plants that form forest canopy layer), non-vascular plants (mosses or lichens), and non-plants (fungi, rotten wood or animal feathers and hairs). Host specificity was considered a factor with four levels and defined as follows: monophagy (only one host species or a maximum of two hosts that belong to the same genus), oligophagy (a minimum of three hosts that belong to the same genus, or a maximum of two closely related host genera), and polyphagy (several host species or a minimum of three host genera). The fourth group consisted of species whose host specialization is unknown. Historical range was defined as species-specific mean elevation value (i.e., center-of-gravity) at the beginning of the surveillance period (1978–1982). Absolute elevation values were standardized prior to analysis by subtracting the sample mean and dividing by the standard deviation in the whole sample. The model included both the first- and the second-order terms of the historical range. The linear term controlled for possible variation among species that would simply emerge due to the fact that species with a high early elevation value would have less space to expand their range upwards compared to species that occur at a lower elevation. The quadratic term was included to control for habitat-specific expansion propensity that the linear term may not necessarily capture. For example, species associated with the Scots pine zone (the second lowest vegetation zone) showing either strong or weak expansion rates relative to species associated with any other vegetation zone would result in negative or positive quadratic terms, respectively. To control for any bias due to variable evolutionary history (i.e., phylogenetic relatedness) among the species, we included Superfamily, Family and Genus a certain species belong to as random factors. Moran’s *I* for different nested levels of taxonomic hierarchy suggests only negligible phylogenetic signal in the species-specific responses [0.29 (Genus)–− 0.04 (Superfamily)] (Online Resource 2), which justifies only inclusion of the robust grouping variables instead of specific metrics of phylogenetic interdependencies. Moran’s *I* was calculated with the function *correlogram.formula* in the R package *Ape 5.2* (Paradis et al. 2018) based on the robust taxonomical hierarchy given by Finnish Biodiversity Info Facility (https://laji.fi).

#### Flight periodic shifts across years

To explore changes in adult flight periods across the years, we first estimated the length of the annual flight periods of a set of species with the most comprehensive data. The group of 57 selected species was reduced to ones with an average yearly abundance of more than 30 individuals. Then, only species with more than 20 such years were selected. Altogether 27 species fulfilled these criteria. To estimate species-specific annual flight periods, we fitted separate GAMs [function *gam* (Wood 2006)] to year-specific phenological data of each species. The annual flight period was estimated as the number of days when [log^10^(*n* + 1)]-transformed average daily catch exceeded 0.2, which corresponds to a daily catch of 1.6 individuals. This threshold was selected to estimate the length of the main flight period neither affected by single exceptionally early nor late individuals.

Temporal variation in flight periods was explored with a GLMM where the estimated annual flight period was set as the response variable. Fixed effects were estimated for year, moth abundance as well as for average temperature and thermal variation during a species-specific flight period and their two-way interaction. Moth abundance was included to control for uncertainty in the estimation of year-specific flight periods arising from occasional small sample sizes and a possible positive relationship between abundance and flight period. The latter could emerge because in peak years exceptionally early or late individuals are more likely detected compared to years when population size is low. Variation in thermal conditions during the flight periods within a year was characterized by coefficient of variation in mean daily temperatures. Random effect structure included only species-specific random intercepts (*υ*_species_ = 0.002, *υ*_residual_ = 0.002).

### Moth assemblages in space and in time

Moth assemblage structure was explored with non-metric multidimensional scaling (NMDS) [function *metaMDS* (Oksanen et al. 2017)]. In describing assemblage structures, each species resident in 1978–1979 (249 species, 234,404 individuals) was included irrespective of their abundance. Each trap was considered an independent replicate sampling site within the vegetation (i.e., elevation) zone it was placed. To explore temporal trends in moth assemblage structures, we arbitrarily divided the sampling period 1978–2012 into nine 4-year periods (the last one 2010–2012: 3 years) that would represent the moth assemblage at that particular sampling site and time. With this procedure, we ended up with a total of 99 [(3 × 3 × 9) + (2 × 9)] assemblages. The 4-year species-specific moth catches at each sampling site were [log^10^(*n *+ 1)]-transformed prior to analysis. The NMDS was first run with the whole data and then for the three lowest habitat types only. To explore correspondence between assemblage configurations with the environment, we fitted environmental variables [elevation of a particular trap (i.e., assemblage), time period (an ordered factor with nine levels) and minimum season length within a particular time period] onto the NMDS configurations with the function envfit (Oksanen et al. 2017).

## Results

### Temporal trends in climate

**Fig. 1:**
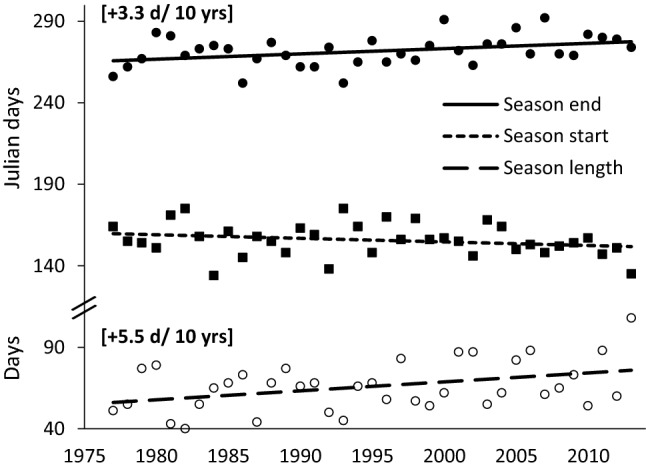
Temporal trends in the three climate variables (season length, season start, season end) in Värriö 1977–2013 [effect sizes given in brackets for statistically significant trends]

The beginning of the favorable season (i.e., “season start”) varied considerably among the years (range: May 14–June 25), but did not become earlier during the period of 1977–2013 (*b* = − 0.2183, S.E. = 0.1518, *t *= − 1.438, *p* = 0.1593) (Fig. 1). The “season length” varied in an order of magnitude among years (range: 40–108 days) and became longer during the monitoring period at the average rate of 5 days per decade (*b* = 0.549, S.E. = 0.216, *t* = 2.538, *p* = 0.016) (Fig. 1). At the same time, the “season end” (range: September 10–October 20) became later at the average rate of 3 days per decade (*b* = 0.3250, S.E. = 0.1353, *t* = 2.402, *p* = 0.0218) (Fig. 1).

### Effects of season length and onset of winter on moth abundance

**Fig. 2:**
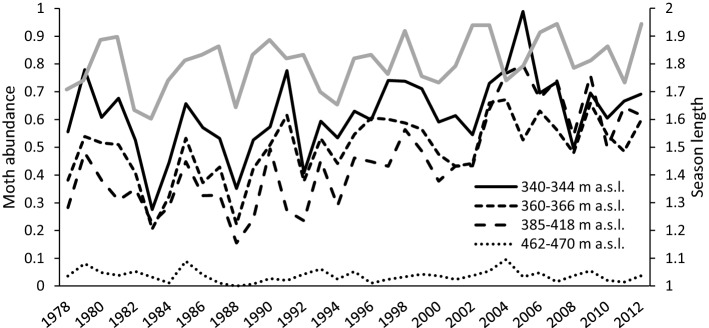
Correspondence between the season length a year before and moth abundance in the following summer at different elevations (i.e., vegetation zones: 340–344 m a.s.l. = spruce-dominated coniferous forest), 360–366 m a.s.l = Scots pine-dominated coniferous forest), 385–418 m a.s.l. = mountain birch-dominated deciduous forest, 462–470 m a.s.l. = treeless tundra)

The total moth catch varied considerably among the years (range: 2238–32,261 individuals), population dynamics of the Autumnal moth (*Epirrita autumnata*) alone being largely responsible of that absolute interannual variation. The length of the preceding season explained variation in moth abundance in general so that moth abundance increased with increasing season length after controlling for species-specific random variation (Table 1, Fig. 2). This suggests that natural variation in the season length has a capacity to constrain realized fitness of moths and thus affect their occurrence in the area. The response was similar at different elevations except for the treeless tundra where moth abundance was invariably low (Table 1). A positive association between late onset winter and moth abundance emerged at the second highest elevation (385–418 m a.s.l.) (Table 1).

**Table 1:** Fixed effects of a GLMM explaining annual variation in moth abundance among the 57 moth species in relation to elevation as well as the length and ending of the previous season

Parameter	Estimate (posterior mean)	95% HPD credibility interval		*N*_eff_
		Lower limit	Upper limit	
Intercept (elevation *a*)	0.41	− 0.76	1.55	4500
Elevation (*b*)	− 0.10	− 0.56	0.37	4288
Elevation (*c*)	− 1.29	− 1.73	− 0.76	4400
Elevation (*d*)	− 0.16	− 0.76	0.37	4500
Season length	2.62 × 10^−3^	8.7 × 10^−5^	4.75 × 10^−3^	4500
Season end	8.35 × 10^−4^	− 3.03 × 10^−3^	4.4 × 10^−3^	4716
Elevation (*b*) × season length	1.87 × 10^−5^	− 9.99 × 10^−4^	1.0 × 10^−3^	4500
Elevation (*c*) × season length	− 2.61 × 10^−4^	− 1.20 × 10^−3^	7.76 × 10^−4^	4500
Elevation (*d*) × season length	− 2.58 × 10^−3^	− 3.63 × 10^−3^	− 1.40 × 10^−3^	4500
Elevation (*b*) × season end	− 3.39 × 10^−5^	− 1.53 × 10^−3^	1.51 × 10^−3^	3731
Elevation (*c*) × season end	4.33 × 10^−3^	2.83 × 10^−3^	5.89 × 10^−3^	4500
Elevation (*d*) × season end	− 6.67 × 10^−4^	− 2.30 × 10^−3^	1.12 × 10^−3^	4500

The model was parameterized so that the lowest elevation [*a* = 340–344 m a.s.l. (spruce-dominated coniferous forest)] forms the reference category to which the higher elevations [*b* = 360–366 m a.s.l (Scots pine-dominated coniferous forest), *c* = 385–418 m a.s.l. (mountain birch-dominated deciduous forest), *d* = 462–470 m a.s.l. (treeless tundra)] are compared. Random effects included individual traps (i.e., repeated measurements within a particular elevation zone) as well as random intercepts for year and species that was correlated with elevation (a continuous variable ranging 0–130 m)

### Temporal trends in species’ elevation changes

**Fig. 3:**
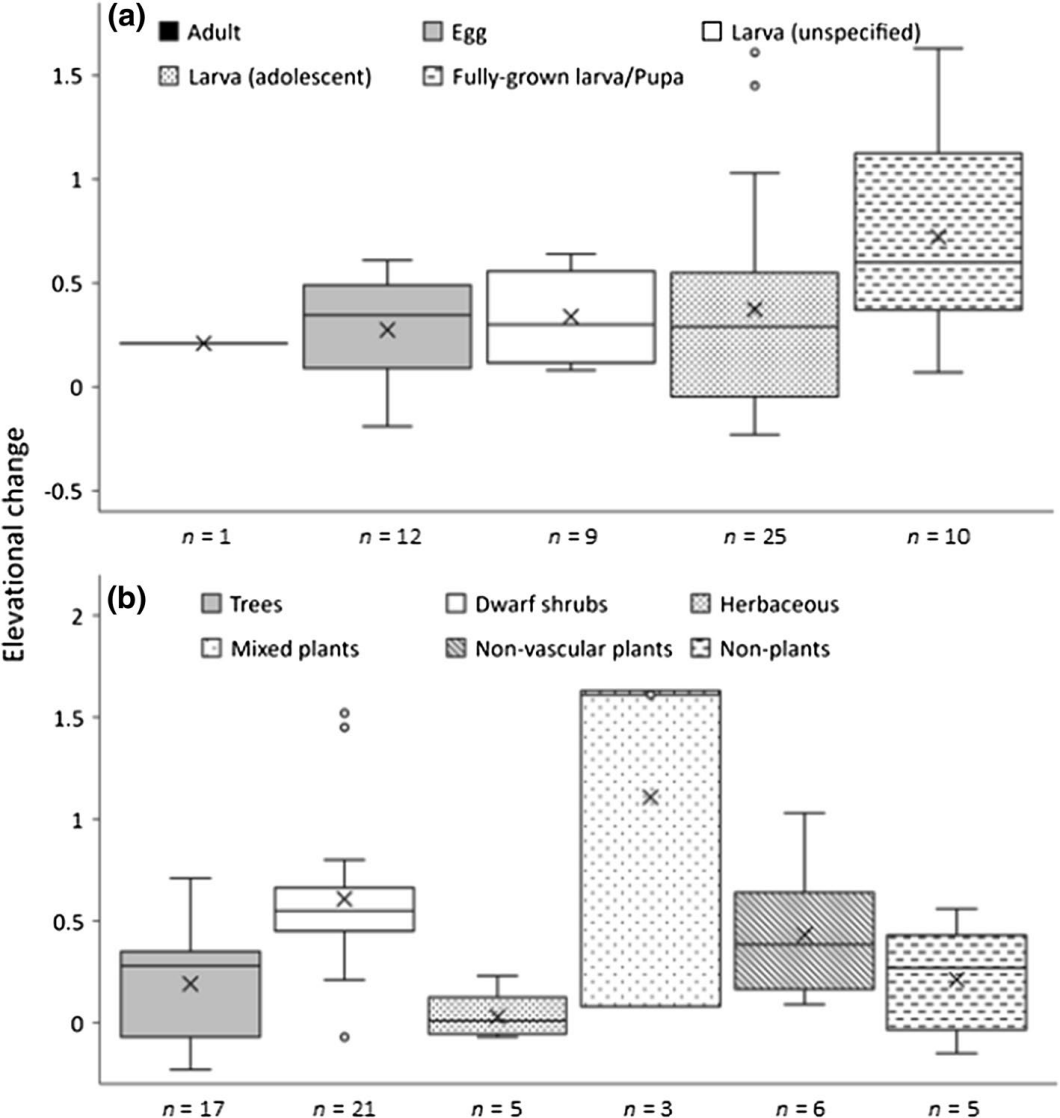
Variation in elevational changes among the groups of species with **a** different over-wintering developmental stage [unspecified larva refers to species that over-winter in the larval stage, but it is not known whether larvae continue feeding after diapause] and **b** host type [× = mean; black line = median; box = the first and the third quartiles; whiskers = minimum and maximum values excluding outliers (open circles)]

The elevational center-of-gravity changed uphill over the course of study in 39 out of the 57 selected species (Table 2). Fifteen species did not show statistically significant trends in time, whereas for three species the elevational center-of-gravity changed downhill (Table 2). Responding and non-responding species as well as species with either increasing or decreasing slope values were found in each larger taxonomic group (Online Resource 1) and phylogeny did not explain variance in slope values (see Table 3). A significant negative quadratic term of the historical elevation value suggests that species originally associated with mid-elevation habitats showed an exaggerated response compared to species that occurred either at low or high elevations in 1978–1981 (Table 3). A significant positive term for the diapause stage “larva (fully grown)/pupa” implies that species completing their growth period in autumn, thus facing the most severe risks of autumn frosts, moved uphill more than an average species that over-winters at any other developmental stage (Table 3, Fig. 3a). Similarly, species that follow a 2-year life cycle expressed lower slope values compared to species that complete their development within a year (Table 3). Species whose larvae dwell on the forest canopy or feed on herbaceous plants were less likely to expand their range compared to species that feed on dwarf shrubs (Table 3, Fig. 3b). A seemingly positive effect of the “mixed diet” [low sample size (*N* = 3), Fig. 3b] was overruled by a marginally non-significant positive term for “monophagy” over the more diverse diets (Table 3). Yet, host specificity per se did not explain variation in slope values among the species.

**Table 2:** Species-specific temporal changes in the elevational center-of-gravity (i.e., posterior mean of correlation between annual elevation values and time, a slope value) based on a GLMM with a fixed intercept and a random effect structure including random intercepts for the species that were correlated with year

Species	Slope	95% HPD interval		Species	Slope	95% HPD interval	
		Lower	Upper			Lower	Upper
*Acleris maccana*	0.21	0.17	0.25	*Mompha idaei*	0.23	− 0.01	0.44
*Ancylis myrtillana*	0.60	0.55	0.65	*Mompha locupletella*	0.02	− 0.15	0.19
*Ancylis unguicella*	1.52	1.27	1.77	*Monopis laevigella*	0.27	− 0.12	0.62
*Argyresthia svenssoni*	0.28	2.0 × 10^−3^	0.53	*Monopis spilotella*	0.56	0.27	0.85
*Bryotropha galbanella*	0.33	0.20	0.47	*Nemapogon cloacellus*	− 0.15	− 0.39	0.11
*Chionodes continuellus*	0.19	0.13	0.26	*Neofaculta infernella*	− 0.07	− 0.20	0.05
*Chionodes nubilellus*	0.09	− 0.08	0.31	*Operophtera brumata*	0.34	0.26	0.42
*Clepsis senecionana*	1.63	1.37	1.88	*Paraswammerdamia conspersella*	0.55	0.52	0.58
*Coleophora glitzella*	0.55	0.40	0.69	*Phiaris bipunctana*	1.45	1.21	1.67
*Coleophora idaeella*	0.54	0.25	0.82	*Phiaris heinrichana*	0.43	0.30	0.57
*Coleophora vacciniella*	0.73	0.52	1.01	*Phiaris obsoletana*	0.64	0.58	0.70
*Denisia similella*	0.30	0.21	0.38	*Phiaris palustrana*	1.03	0.85	1.19
*Denisia stipella*	0.08	0.03	0.15	*Phiaris schulziana*	− 0.50	− 0.55	− 0.45
*Dystroma citratum*	0.08	− 8.0 × 10^−3^	0.18	*Pleurota bicostella*	0.55	0.47	0.62
*Eana osseana*	1.61	1.51	1.70	*Poecilocampa populi*	− 0.19	− 0.29	− 0.01
*Ectoedemia weaveri*	0.46	0.36	0.59	*Pseudatemelia josephinae*	− 0.17	− 0.38	0.06
*Elachista parasella*	0.01	− 0.16	0.18	*Scopula ternata*	0.42	0.32	0.53
*Elophos vittarius*	0.44	0.32	0.56	*Selenia dentaria*	0.30	0.14	0.46
*Entephria caesiata*	0.27	0.24	0.29	*Sparganothis rubicundana*	0.80	0.67	0.92
*Epinotia maculana*	0.35	0.24	0.46	*Stigmella lapponica*	0.71	0.45	0.94
*Epinotia solandriana*	0.12	− 6.0 × 10^−3^	0.32	*Syngrapha interrogationis*	− 0.23	− 0.36	− 0.11
*Epirrita autumnata*	0.37	0.36	0.39	*Taleboria borealis*	0.44	0.24	0.64
*Eudonia murana*	0.51	0.30	0.69	*Thera obeliscata*	0.42	0.24	0.62
*Eulia ministrana*	0.07	− 0.12	0.27	*Trichiura crataegi*	− 0.07	− 0.27	0.14
*Eulithis populate*	0.53	0.49	0.56	*Xanthorhoe decoloraria*	− 0.07	− 0.24	0.01
*Eulithis testata*	0.61	0.38	0.87	*Ypsolopha parenthesella*	0.22	0.09	0.37
*Eupithecia pusillata*	0.35	0.20	0.48	*Zeiraphera griseana*	0.42	0.26	0.59
*Hellinsia tephradactyla*	− 0.04	− 0.34	0.29	Increased	39 species		
*Incurvaria vetulella*	0.10	− 0.02	0.23	Stable	15 species		
*Lithomoia solidaginis*	0.58	0.51	0.65	Decreased	3 species

**Table 3:** Fixed effects of a GLMM explaining variation in species-specific uphill change in center-of-gravity in relation to historical range (COG1978 = standardized mean center-of-gravity in 1978–1982), over-wintering developmental stage (adult; egg; adolescent larva; larva in a unspecified developmental stage; fully grown larva or pupa), life cycle (annual; biennial), host type (dwarf shrubs; herbaceous plants; trees and bushes = canopy; mixed diet = woody dwarf shrubs and herbaceous plants; non-vascular plants = mosses or lichens; non-plants = rotten wood, fungi or animal feathers and hairs), and the degree of host specificity (polyphagy; oligophagy; monophagy; unspecified)

Source of variation	Parameter	Estimate (posterior mean)	95% HPD credibility interval		*N*_eff_
			Lower limit	Upper limit	
	(Intercept)	10.18	− 11.3	31.81	> 20,000
Historical range	COG1978	0.50	− 0.02	1.22	> 20,000
	(COG1978)^2^	− 0.02	− 0.03	− 1.65 × 10^−4^	> 20,000
Over-wintering stage (adult)	Egg	18.08	− 3.70	39.70	> 20,000
	Larva (unspecified)	19.45	− 3.02	42.13	> 20,000
	Larva (adolescent)	18.15	− 2.91	39.12	> 20,000
	Larva (fully grown)/pupa	26.95	5.47	48.04	> 20,000
Host type (dwarf shrubs)	Herbaceous plants	− 28.90	− 40.76	− 17.32	> 20,000
	Mixed diet	1.14	− 14.13	12.20	> 20,000
	Trees or bushes	− 14.34	− 22.04	− 6.51	> 20,000
	Non-vascular plants	− 0.63	− 14.59	13.52	> 20,000
	Non-plants	− 14.44	− 29.92	1.03	> 20,000
Life cycle (annual)	Biennial	− 12.37	− 23.78	− 1.17	13,732
Host specificity (polyphagy)	Monophagy	6.73	− 0.015	13.49	> 20,000
	Oligophagy	− 1.83	− 10.15	6.40	> 20,000
	Unspecified	− 2.95	− 15.45	9.22	> 20,000

Random effect variances: superfamily: 3.53 (1.40 × 10^−4^..0.18.25), family: 5.33 (1.20 × 10^−4^…28.5), genus: 36.99 (1.04 × 10^−4^..0.109.80), model residuals: 57.36 (8.05…112.00)

Random effect structure included grouping factors genus, family and superfamily to control for varying evolutionary history of species

### Temporal trends in flight periods

There was no temporal trend in the length of adult flight periods among the studied 27 moth species after controlling for variation in moth abundance (Table 4). Variation in flight periods was explained by interaction between mean ambient temperature and variability of thermal conditions during the adult phase (Table 4). A high mean temperature shortened the flight period of a species, but the effect was mitigated by high daily fluctuations in thermal conditions that have a tendency to prolong the flight period.

**Table 4:** Fixed effects of a GLMM explaining variation in the annual flight periods among the 27 moth species in relation to time [year] and weather conditions during the adult stage [mean/coefficient of variation]

Parameter	Estimate (posterior mean)	95% HPD credibility interval		N_eff_
		Lower limit	Upper limit	
(Intercept)	− 37.29	− 139.77	67.62	> 200,000
Year	0.04	− 0.01	0.10	> 200,000
Abundance	5.38	4.35	6.43	> 200,000
Temperature_mean_	− 2.00	− 2.39	− 1.60	> 200,000
Temperature_cv_	− 5.17	− 10.67	0.49	> 200,000
Temperature_mean_ × temperature_cv_	2.52	1.58	3.45	> 200,000

Random effects included species-specific random intercepts. Random effect variances: species: 35.10 (16.68…57.38), model residuals: 58.70 (53.15…64.27)

### Moth communities in space and in time

**Fig. 4:**
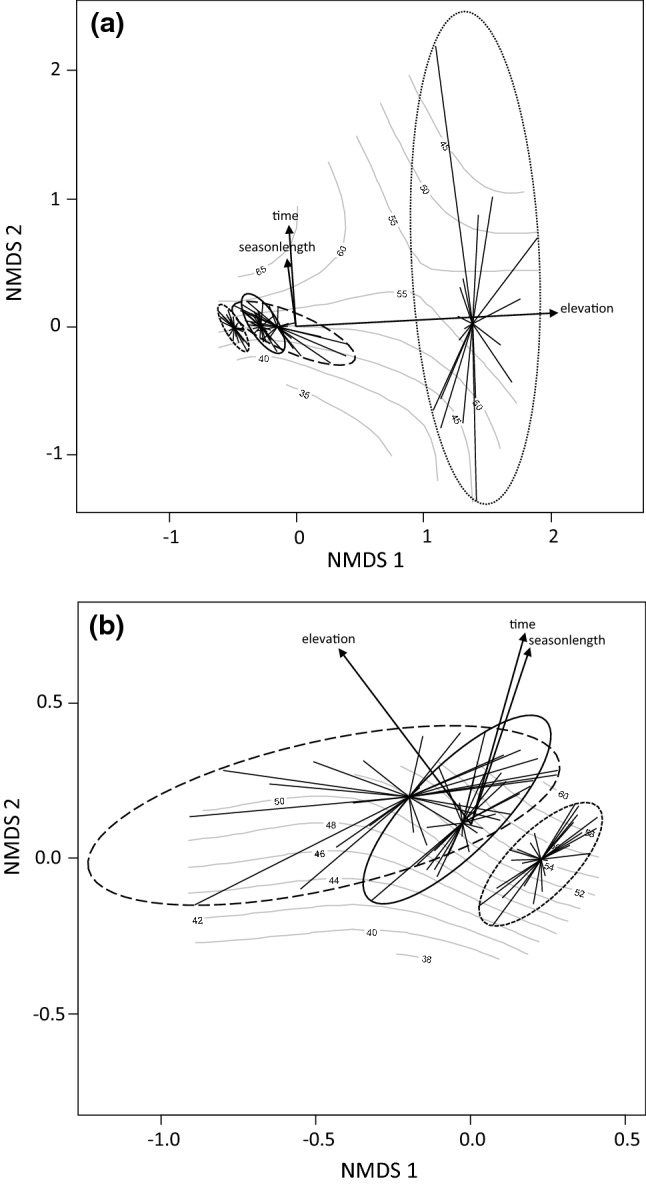
Two-dimensional NMDS configurations of moth assemblages (ellipsoid hull and spider web diagrams) plotted on fitted thin plate spline surfaces in relation to the minimum season length: **a** each elevation zone included, **b** only the three lowest elevation zones included [short dash line = spruce-dominated coniferous forest (340–344 m a.s.l.), solid line = Scots pine-dominated coniferous forest (360–366 m a.s.l), long dash line = mountain birch-dominated deciduous forest (385–418 m a.s.l.), dotted line = treeless tundra (462–470 m a.s.l.)]. Tips of the lines represent a certain assemblage and the respective line its distance to an average assemblage within a particular elevation zone. Black arrows represent the main directions of variation in assemblage structures in relation to the minimum season length, time and elevation, while arrow lengths illustrate the strength of the respective correlations

Moth assemblages at the two highest sampling sites located in the treeless tundra differed from those at the lower altitude sampling sites located in forested habitats (Fig. 4a). Elevational gradient was strong in the direction of the first NMDS axis (NMDS 1: 0.99; NMDS 2: 0.05; *r*^*2*^ = 0.76; *p* < 0.001), whereas time from the beginning of the moth monitoring and the minimum season length correlated to a lesser extent with the second NMDS axis (time: NMDS 1: − 0.08; NMDS 2: 0.99; *r*^*2*^ = 0.11; *p* < 0.003; season length: NMDS 1: − 0.14; NMDS 2: 0.99; *r*^*2*^ = 0.08; *p* < 0.072). There was more variation in assemblage structure in time among tundra assemblages than among assemblages at forested sites. A fitted ordination surface reveals that variation among tundra assemblages is highly stochastic in relation to season length compared to assemblages of the forested sites.

**Fig. 5:**
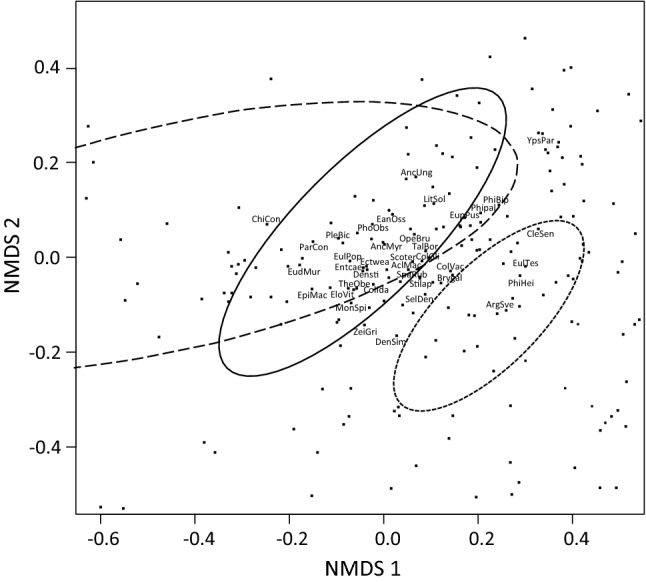
Associations of individual moth species with certain habitat-specific moth assemblages [short dash line = spruce-dominated coniferous forest (340–344 m a.s.l.), solid line = Scots pine-dominated coniferous forest (360–366 m a.s.l), long dash line = mountain birch-dominated deciduous forest (385–418 m a.s.l.)] according to a two-dimensional NMDS configuration. Species that showed temporal changes in the elevational center-of-gravity are abbreviated (the three first letters of genus and species names, see Table 2 for the whole binomial names), whereas the others are only given as dummy numbers

Exclusion of the tundra sites indicated that any variation among moth assemblages of the different forested vegetation zones can be explained by the elevational gradient (NMDS 1: − 0.60; NMDS 2: 0.80; *r*^*2*^ = 0.77; *p* < 0.001). The main direction of variation within a certain vegetation zone (spruce and pine forests, in particular) was almost parallel to the time and season length axes indicating season length-dependent temporal trends in assemblage structures (time: NMDS 1: 0.28; NMDS 2: 0.96; *r*^*2*^ = 0.63; *p* < 0.001; season length: NMDS 1: 0.32; NMDS 2: 0.95; *r*^*2*^ = 0.56; *p* < 0.001) (Fig. 4b). Moreover, direction of the season length axis together with the fitted surface reveals that moth assemblages at the mountain birch zone have changed and become practically inseparable from those at the Scots pine zone as the season length has increased. This is because the species whose elevational center-of-gravity had changed uphill were mostly species originally associated with the Scots pine-dominated vegetation zone but expanded their range into the Mountain birch-dominated zone during the period 1978–2012 (Fig. 5). In contrast, only three species associated with spruce forest had changed uphill.

## Discussion

The length of the favorable season for insect growth and development has increased and the onset of winter has delayed in Värriö over the years 1977–2013. Moth abundance and the season length a year before were positively correlated in forest habitats, but not necessarily in the treeless summit of the Värriötunturi fell. A remarkable proportion of moth species shows an upward trend on the elevational gradient over time. Flight periods of the moths did not prolong in time, but were determined by the proximate weather conditions within a certain year. The most prominent upward changes were observed among species that complete their resource acquisition before over-wintering within a season and were associated with pine heat forest. At the community level, moth assemblages in the pine and mountain birch forests have become inseparable, whereas assemblages in the spruce forest and treeless tundra have remained characteristic despite a remarkable year-to-year variation in the latter.

Climatic effects of the global climate warming vary geographically, climatic change becoming somewhat exaggerated towards the poles. The freeze-free periods in high latitudes are lengthening with a concomitant decrease in snow cover (Walther et al. 2002). Changes in the precipitation are neither spatially nor temporally uniform. Increases in autumn and/or winter precipitation have been reported in high latitudes, whereas precipitation tends to decrease in the sub-tropics (Walther et al. 2002). In our boreal study site, significant increases in spring, autumn, and winter temperatures, and in winter precipitation have been reported (Hunter et al. 2014). Our analysis adds a prolonged favorable season among the climate variables that show significant temporal change. The pattern emerged mostly as a response to onsets of winters occurring later (an increase in autumn temperatures) and to a lesser extent to advancing springs. The favorable season has become longer at a rate of 5 days per decade, which means a 34% increase in the season length (57 vs. 76 days) over the course of the monitoring period. Such a change is unlikely trivial for organisms whose development depends on external sources of heat, such as insects. Accordingly, the season length a year before appeared positively correlated with moth abundance in the following season. The increasing season length and its seemingly deterministic effects on moth abundance explains well the reported overall temporal increase in moth abundance in Värriö (Hunter et al. 2014, cf. Fig. 2), which contradicts a recent finding of dramatic decrease of insects in the temperate zone (Hallmann et al. 2017). More importantly, the correlation withstands theoretical investigations (Roff 1980; Iwasa et al. 1994; Kivelä et al. 2013) that emphasize the importance of season length in determining insect fitness. In addition, the positive association of the late onset of winter and moth abundance close to the timber line further stresses the need to reach a species-specific over-wintering stage before the autumn frosts (Tauber et al. 1986).

A taxonomically wide range of organisms have reacted to climate warming or derived environmental changes by shifting or expanding their distributions to higher latitudes and/or altitudes (Parmesan 2006; Walther et al. 2002; Fält-Nordmann et al. 2018). Unlike for plants or vegetation (e.g., Kullman 2002; Walther et al. 2002; Peñuelas and Boada 2003; Franke et al. 2019), unambiguous empirical evidence of insects shifting to higher altitudes with demonstration of causality is few (but see Wilson et al. 2005; Franco et al. 2006; Merrill et al. 2008). The missing evidence is probably due to a lack of adequate long-term data as changes in species’ distributions are expected particularly when elevational gradients allow organisms to track changing conditions over reasonably short distances (Pounds et al. 1999; Hill et al. 2002). Accordingly, we observed that 39 (68%) of the selected 57 boreal moths had increased or expanded upwards on the elevational gradient in Värriötunturi fell from the late 1970s. The species with different responses distributed evenly among groups of species that face different light conditions (see Online Resource 1). Accordingly, the general pattern is unlikely driven by sampling bias due to increasingly suitable flight conditions in late summer (higher trapping efficiency due to longer dark period). This corresponds closely to observations of 68% geometrid moths (*N* = 102) showing increases in their center-of-gravity in Mt. Kinabalu in Borneo (Chen et al. 2009) and 73% butterfly species (*N* = 19) in Sierra de Guadarrama in Spain (Wilson et al. 2005) within a comparable time. In Sierra de Guadarrama, local extinctions at low elevation margins rather than changes in upper elevational limits explained the increase in center-of-gravity, which contradict the mechanism in Värriö based only on relative changes in abundance at different elevations. Nevertheless, the observed correlation between season length and moth abundance offers a causal link not only for the expanding cool range margins, but may equally apply also to contractions of warm margins where a changing thermal environment has a capacity to result in mismatches between season length and phenology of essential life history events (see Van Dyck et al. 2015).

Herbivorous insects cannot readily expand beyond ranges of certain host plants although the prevailing climate would sustain population persistence (Merrill et al. 2008; but see Pateman et al. 2012). Thus, generalists have been considered as the main beneficiaries of climate warming (Warren et al. 2001; Menéndez et al. 2006, 2007; González-Megías et al. 2006, 2008; Pöyry et al. 2009; Clavel et al. 2010). In our case, the degree of host specificity per se did not explain variation in slope values among the species. There was, however, a tendency of the monophagous species to show the strongest response, which simply reflects the fact that host plants of such moth species in our sample, like *Vaccinium vitis*-*idaea*, *Calluna vulgaris* and *Betula pubescens* are abundant and occur everywhere in the landscape. The former two plant species also reflect a more general tendency of species associated with dwarf shrubs readily expanding their range uphill. Dwarf shrubs are known to respond to climate change and expand their range (Kullman 2002; Walther et al. 2002). We have no data on possible changes in vegetation in Värriö. Yet, shrubs utilized by the moth species are invariably abundant and thus a more likely explanation for the uphill range expansions is the changing climatic envelope. In accordance with the climatic explanation grounded on the assumption that season length constrains insect fitness, species that complete acquisition of resources used for reproduction before over-wintering within a season and thus face the most severe seasonal time constraints were the ones taking advantage of the changing climate the most. This is applicable to a majority of nocturnal moths that over-winter in the pupal stage or as a full-grown larva, but not necessarily to species with 2-year life cycle or species whose reproductive potential depends on adult feeding such as many butterflies (see Pöyry et al. 2009). To explicitly test for the latter hypothesis, data that include species covering the whole capital vs. income breeding continuum would be needed.

The theoretical predictions of prolonged adult lifespan and high fecundity as a response to relaxing seasonal time constraints (Kivelä et al. 2013) would be attributable to a prolonged flight period of a particular species. Although the underlying premise of relaxing time constraints held true, flight periods of the 27 most abundant species did not change over time in Värriö. Thus, this mechanism cannot explain increasing overall moth abundance in the area (Hunter et al. 2014). The lack of temporal change may reflect a fact that natural selection acts via ultimate factors, but the proximate environment determines the expressed phenotype for selection to act on (Ghalambor et al. 2007). Proximate weather conditions during the adult phase determined the length of flight periods among the moth species. High ambient temperatures resulted in relatively short flight periods, whereas highly fluctuating daily temperatures tended to prolong them. This being the case, any temporal trends would reflect changes in the proximate environment, while phenotypic plasticity due to temperature dependence of insect physiology (see Angilletta 2009) would mask possible variation among genotypes and hinder evolutionary responses. This is not to say that selection would not underlie the reported prolongations of flight periods among Lepidoptera that at least partly arise from increases in voltinism (Roy and Sparks 2000; Pöyry et al. 2011). Selection readily favors multivoltinism as long as the favorable season becomes sufficiently long (Roff 1980; Iwasa et al. 1994; Kivelä et al. 2013) and in addition to environmental determination, variation in the degree of voltinism has a genetic component among Lepidoptera (Välimäki et al. 2008; Kivelä et al. 2012; Välimäki et al. 2013a, b).

Habitat and/or host plant associations partly explain the curvilinear trend of species at middle elevations expressing the strongest expansion upwards. For obvious reasons, species that prefer open tundra did not show any increase in their center-of-gravity, while species tightly associated with Norwegian spruce forest are necessarily constrained to low elevations. The pronounced responses among species originally associated with the pine heat forest at middle elevation resulted in a merged moth assemblage of pine and mountain birch zones on the elevational gradient. The change is of similar magnitude like mean uphill shifts of Geometrid assemblages (41.9 m) in Mt. Kinabalu (Chen et al. 2009). Such a modification of insect assemblages due to upward range expansion is not exceptional elsewhere either (González-Megías et al. 2008; Zografou et al. 2014; Nieto-Sánchez et al. 2015). We, however, stress that the turnover of species assemblages in Värriö was completely due to changes in species abundance, as we considered only species that were resident already in the late 1970s and none of the species became extinct over the course of the study. Our results imply that if host plants do not constrain occurrence, assemblages that consist of habitat specialist are less likely to show temporal trends compared to assemblages dominated by species with loose habitat preferences. Accordingly, Nieto-Sánchez et al. (2015) have suggested that habitat-specific biotic interactions constrain species assemblages from responding to environmental change. The moth assemblage in the spruce forest zone changed somewhat over time but remained characteristic overall. The same applies to the moth community in the treeless tundra, except that short-term random turnover of the assemblage structure was evident. This probably mirrors a variable degree of environmental stochasticity (Martin 2001). In the forested areas, tree canopy mitigates environmental uncertainty and results, for example, in relatively stable snowpack and snowmelt in winter–spring as well as in stable temperatures, humidity and wind conditions in summertime. In the treeless tundra, a lack of tree cover translates to stochastic variation in the above-mentioned variables that are known to affect the habitable climatic envelope of phytophagous insects (e.g., Menéndez et al. 2007; Merrill et al. 2008). We, however, acknowledge that the rapid turnover of moth assemblages in tundra may partly arise due to low numbers of species and individuals involved, which likely increases sampling error.

To conclude, the length of the favorable season apparently constrains fitness of certain insects. Easing seasonal time constraints due to climate change enables such species to expand their distributions on elevational gradients. This applies to species that are generalists or associated with common host plants and thus relatively free of restrictive biotic interactions with their host plants. At the assemblage level, different proximate factors determine temporal resilience at different timescales. Strong biotic interactions buffer assemblages against long-term directional environmental change, whereas short-term environmental variation results in stochastic assemblage dynamics that obscure long-term patterns. Nevertheless, long-term changes in species assemblages modify biotic interactions. Direct interactions (i.e., competition) among herbivorous insects is unlikely important because plant productivity usually exceeds herbivory except occasionally within the boreal populations of the Autumnal moth (*Epirrita autumnata*) or the Winter moth (*Operophtera brumata*) (Tenow et al. 2007). Indirect interactions mediated by shared parasitoids or predators at a higher trophic level, in turn, may profoundly affect moth population dynamics (Klemola et al. 2009). At the landscape scale, simultaneously expanding cool margins of several moth species results in diverse and stable larval assemblages widely distributed in time within a season, which is likely beneficial for insectivorous birds whose reproductive output depends on food availability during nestling (Visser et al. 2006) and/or post-nestling (Pakanen et al. 2015) phases of the reproductive cycle.

## Electronic supplementary material

Below is the link to the electronic supplementary material.

Supplementary material 1 (DOCX 34 kb)

Supplementary material 2 (DOCX 29 kb)
