# Supplementary material for: Climate change-driven elevational changes among boreal nocturnal moths
Source: Oecologia. 2020 Apr 8;192(4):1085–98. doi: 10.1007/s00442-020-04632-w (PMC7165148; doi:10.1007/s00442-020-04632-w)
Supplement: Supplementary file 1 — Supplementary material 1 (DOCX 34 kb) [file 442_2020_4632_MOESM1_ESM.docx]

Online Resource 1.

Keret NM, Mutanen MJ, Orell MI, Itämies JH, Välimäki PM. Climate change driven elevational changes among boreal nocturnal moths

**Life history and ecological properties of the moth species included in the analyses**

| Family | Species (total catch) | ^†^Pheno-logy | Elevational change | Standardized center-of-gravity 1978 | Over-wintering stage | Life-cycle | Host type | Host specificity | Main hosts |
| --- | --- | --- | --- | --- | --- | --- | --- | --- | --- |
| Coleophoridae | *C. glitzella* (1194) | mid | 0.55 | -0,42 | larva (adolescent) | annual | dwarf shrubs | oligophagous | *Vaccinium* |
|  | *C. idaeella* (287) | mid | 0.54 | -0.08 | larva (adolescent) | annual | dwarf shrubs | monophagous | *Vaccinium vitis-idaea* |
|  | *C. vacciniella* (282) | mid | 0.73 | -0.41 | larva (fully-grown) | annual | dwarf shrubs | oligophagous | *Vaccinium* |
| Elachistidae | *E. parasella* (707) | mid | 0.01 | -1.56 | larva (adolescent) | annual | herbaceous | polyphagous | *Carex* |
| Gelechiidae | *B. galbanella* (999) | mid | 0.33 | -0.13 | larva (unspecified) | annual | non-vascular | unspecified | mosses |
|  | *C. continuellus* (3753) | mid | 0.19 | 1.79 | larva (unspecified) | annual | non-vascular | polyphagous | lichenes |
|  | *C. nubilellus* (499) | mid | 0.09 | 1.27 | larva (unspecified) | annual | non-vascular | unspecified | mosses? |
|  | *N. infernella* (1987) | mid | -0.07 | 1.08 | larva (adolescent) | annual | dwarf shrubs | oligophagous | *Vaccinium, Betula* |
| Geometridae | *D. citratum* (7591) | late | 0.08 | -0.65 | egg | annual | mixed plants | polyphagous | *Vaccinium, Rubus, Betula* |
|  | *E. vittarius* (1650) | mid | 0.44 | 0.44 | larva (fully-grown) | biennial | dwarf shrubs | polyphagous | *Vaccinium, Betula* |
|  | *E. caesiata* (28817) | late | 0.27 | -0.33 | larva (adolescent) | annual | dwarf shrubs | polyphagous | *Vaccinium, Betula, Ledum* |
|  | *E. autumnata* (151572) | late | 0.37 | 0.12 | egg | annual | trees / bushes | polyphagous | *Betula, Salix* |
|  | *E. populata* (20468) | late | 0.53 | -0.11 | egg | annual | dwarf shrubs | polyphagous | *Vaccinium, Betula, Populus* |
|  | *E. testata* (306) | late | 0.61 | -0.5 | egg | annual | dwarf shrubs | polyphagous | *Vaccinium, Calluna, Salix* |
|  | *E. pusillata* (1687) | late | 0.35 | -0.1 | egg | annual | trees / bushes | monophagous | *Juniperus communis* |
|  | *O. brumata* (4089) | late | 0.34 | -0.3 | egg | annual | trees / bushes | polyphagous | *Betula, Salix, Prunus* |
|  | *S. ternata* (2118) | mid | 0.42 | 0.46 | larva (adolescent) | annual | dwarf shrubs | oligophagous | *Vaccinium, Calluna* |
|  | *S. dentaria* (977) | early | 0.3 | 0.6 | pupa | annual | trees / bushes | polyphagous | *Betula, Salix, Populus* |
|  | *T. obeliscata* (556) | late | 0.42 | 0.09 | larva (adolescent) | annual | trees / bushes | monophagous | *Pinus sylvestris* |
|  | *X. decoloraria* (627) | mid | -0.07 | -1.37 | larva (adolescent) | annual | herbaceous | monophagous | *Galium* |
| Incurvariidae | *I. vetulella* (1238) | early | 0.1 | -0.62 | larva (adolescent) | biennial | trees / bushes | monophagous | *Betula pubescens* |
| Lasiocampidae | *P. populi* (2889) | late | -0.19 | 1.69 | egg | annual | trees / bushes | polyphagous | *Salix, Populus, Betula,* |
|  | *T. crataegi* (517) | late | -0.07 | 3.61 | egg | annual | trees / bushes | polyphagous | *Salix, Sorbus, Populus* |
| Momphidae | *M. idaei* (395) | mid | 0.23 | -0.35 | larva (adolescent) | annual | herbaceous | monophagous | *Epilob. angustifolium* |
|  | *M. locupletella* (730) | mid | 0.02 | -1.64 | larva (adolescent) | annual | herbaceous | monophagous | *Epilobium palustre* |
| Nepticulidae | *E. weaveri* (1609) | mid | 0.46 | -0.02 | larva (adolescent) | annual | dwarf shrubs | monophagous | *Vaccinium vitis-idaea* |
|  | *S. lapponica* (312) | early | 0.71 | -1.16 | pupa | annual | trees / bushes | monophagous | *Betula pubescens* |
| Noctuidae | *S. interrogationis* (1730) | late | -0.23 | 0.53 | larva (adolescent) | annual | trees / bushes | oligophagous | *Salix, Vaccinium* |
|  | *L. solidaginis* (5818) | late | 0.58 | -0.19 | egg | annual | dwarf shrubs | oligophagous | *Vaccinium, Salix* |
| Oecophoridae | *P. josephinae* (445) | mid | -0.17 | -0.54 | larva (adolescent) | annual | trees / bushes | polyphagous | *Populus, Betula, Vaccinium* |
|  | *D. similella* (3774) | mid | 0.3 | -1.23 | larva (adolescent) | annual | non-plant | unspecified | rotten wood, fungi |
|  | *D. stipella* (4522) | mid | 0.08 | 0.12 | larva (unspecified) | annual | non-plant | unspecified | rotten wood |
|  | *P. bicostella* (2473) | mid | 0.55 | 0.81 | larva (unspecified) | annual | dwarf shrubs | monophagous | *Calluna vulgaris* |
| Psychiidae | *T. borealis* (483) | mid | 0.44 | 0.2 | larva (fully-grown) | annual | non-vascular | polyphagous | mosses, lichenes |
| Pterophoridae | *H. tephradactyla* (213) | mid | -0.04 | -0.43 | larva (adolescent) | annual | herbaceous | monophagous | *Solidago virgaurea* |
| Pyralidae | *E. murana* (558) | mid | 0.51 | 1.48 | pupa | annual | non-vascular | unspecified | mosses |
| Tineidae | *M. laevigella* (144) | mid | 0.27 | 0.34 | larva (unspecified) | annual | non-plant | monophagous | corpses, bird nests |
|  | *M. spilotella* (263) | late | 0.56 | -0.36 | larva (unspecified) | annual | non-plant | monophagous | corpses, bird nests |
|  | *N. cloacella* (367) | mid | -0.15 | -0.39 | larva (adolescent) | annual | non-plant | unspecified | fungi |
| Tortricidae | *A. maccana* (6550) | early; late | 0.21 | -0.4 | adult | annual | dwarf shrubs | polyphagous | *Vaccinium, Ledum* |
|  | *A. myrtillana* (11846) | mid | 0.6 | -0.1 | larva (fully-grown) | annual | dwarf shrubs | oligophagous | *Vaccinium* |
|  | *A. unguicella* (622) | early | 1.52 | 0.05 | larva (fully-grown) | annual | dwarf shrubs | monophagous | *Calluna vulgaris* |
|  | *C. senecionana* (397) | early | 1.63 | -1.36 | larva (fully-grown) | annual | mixed_plants | polyphagous | *Vaccinium, Tanacetum, Picea, Pinus* |
|  | *E. osseana* (4024) | mid | 1.61 | -0.13 | larva (adolescent) | annual | mixed_plants | polyphagous |  |
|  | *E. maculana* (3154) | late | 0.35 | -0.1 | egg | annual | trees / bushes | monophagous | *Populus tremula* |
|  | *E. solandriana* (1175) | late | 0.12 | -0.34 | egg | annual | trees / bushes | polyphagous | *Betula, Salix* |
|  | *E. ministrana* (1268) | mid | 0.07 | -0.29 | larva (fully-grown) | annual | trees / bushes | polyphagous | *Salix, Betula, Alnus* |
|  | *P. bipunctana* (714) | mid | 1.45 | -0.12 | larva (adolescent) | annual | dwarf shrubs | oligophagous | *Vaccinium, Betula nana* |
|  | *P. heinrichana* (1140) | mid | 0.43 | -1.14 | larva (adolescent) | biennial | dwarf shrubs | monophagous | *Vaccinium myrtillus* |
|  | *P. obsoletana* (9280) | mid | 0.64 | 0.96 | larva (unspecified) | annual | dwarf shrubs | polyphagous | *Vaccinium, Arctostaphylos* |
|  | *P. palustrana* (1092) | mid | 1.03 | -0.3 | larva (adolescent) | annual | non-vascular | not_specified | mosses |
|  | *P. schulziana* (12563) | mid | -0.5 | 3.15 | larva (unspecified) | biennial | dwarf shrubs | oligophagous | *Calluna, Vaccinium* |
|  | *S. rubicundana* (1418) |  | 0.8 | -0.59 | larva (adolescent) | annual | dwarf shrubs | polyphagous | *Vaccinium, Arctostaphylos* |
|  | *Z. griseana* (1084) | late | 0.42 | -0.43 | larva (adolescent) | biennial | trees / bushes | monophagous | *Picea abies* |
| Argyresthiidae | *A. svenssoni* (418) | mid | 0.28 | -0.83 | larva1 | annual | trees / bushes | monophagous | *Picea abies* |
| Yponomeutidae | *P. conspersella* (19109) | mid | 0.55 | 1.09 | larva (adolescent) | annual | dwarf shrubs | monophagous | *Empetrum nigrum* |
| Ypsolophidae | *Y. parenthesella* (11649) | late | 0.22 | -0.84 | egg | annual | trees / bushes | monophagous | *Betula pubescens* |

^†^early = 20-May – 15-July (daylength ≈ 24 h), mid = 01-July – 31-July (daylength 24 h – 19 h 16 min), late = 01-August – 30-September (daylength 19 h 16 min – 11 h 14 min).
