# Supplementary material for: Climate change-driven elevational changes among boreal nocturnal moths
Source: Oecologia. 2020 Apr 8;192(4):1085–98. doi: 10.1007/s00442-020-04632-w (PMC7165148; doi:10.1007/s00442-020-04632-w)
Supplement: Supplementary file 2 — Supplementary material 2 (DOCX 29 kb) [file 442_2020_4632_MOESM2_ESM.docx]

Online Resource 2.

Keret NM, Mutanen MJ, Orell MI, Itämies JH, Välimäki PM. Climate change driven elevational changes among boreal nocturnal moths

**Moran’s *I* at different levels of taxonomical hierarchy**
